# A prospective study of grey matter and cognitive function alterations in chemotherapy-treated breast cancer patients

**DOI:** 10.1186/2193-1801-3-444

**Published:** 2014-08-19

**Authors:** Chris Lepage, Andra M Smith, Jeremy Moreau, Emily Barlow-Krelina, Nancy Wallis, Barbara Collins, Joyce MacKenzie, Carole Scherling

**Affiliations:** School of Psychology, University of Ottawa, Vanier Hall, 136 Jean Jacques Lussier, Ottawa, ON K1N 6 N5 Canada; Ottawa Hospital, Civic Campus, 1053 Carling Avenue, Ottawa, ON K1Y 4E9 Canada; Memory and Aging Center, Neurology, UCSF, Sandler Neuroscience Center, 675 Nelson Rising Lane, San Francisco, CA 94158 USA

**Keywords:** Breast cancer, Voxel-based morphometry, Chemotherapy, Cognition, MRI, Neuroimaging

## Abstract

**Purpose:**

Subsequent to chemotherapy treatment, breast cancer patients often report a decline in cognitive functioning that can adversely impact many aspects of their lives. Evidence has mounted in recent years indicating that a portion of breast cancer survivors who have undergone chemotherapy display reduced performance on objective measures of cognitive functioning relative to comparison groups. Neurophysiological support for chemotherapy-related cognitive impairment has been accumulating due to an increase in neuroimaging studies in this field; however, longitudinal studies are limited and have not examined the relationship between structural grey matter alterations and neuropsychological performance. The aim of this study was to extend the cancer-cognition literature by investigating the association between grey matter attenuation and objectively measured cognitive functioning in chemotherapy-treated breast cancer patients.

**Methods:**

Female breast cancer patients (*n* = 19) underwent magnetic resonance imaging after surgery but before commencing chemotherapy, one month following treatment, and one year after treatment completion. Individually matched controls (*n* = 19) underwent imaging at similar intervals. All participants underwent a comprehensive neuropsychological battery comprising four cognitive domains at these same time points. Longitudinal grey matter changes were investigated using voxel-based morphometry.

**Results:**

One month following chemotherapy, patients had distributed grey matter volume reductions. One year after treatment, a partial recovery was observed with alterations persisting predominantly in frontal and temporal regions. This course was not observed in the healthy comparison group. Processing speed followed a similar trajectory within the patient group, with poorest scores obtained one month following treatment and some improvement evident one year post-treatment.

**Conclusion:**

This study provides further credence to patient claims of altered cognitive functioning subsequent to chemotherapy treatment.

## Background

Patient reports of cognitive changes subsequent to chemotherapy exposure abound in the breast cancer population. Self-perceived deterioration in mental functioning can adversely impact work and family life for breast cancer survivors (Boykoff et al. 2009). Evidence of chemotherapy-related cognitive impairment (CRCI) in breast cancer patients has mounted in the last several decades, as both retrospective cross-sectional and prospective longitudinal neuropsychological studies have found varying degrees of cognitive under-performance in chemotherapy-exposed breast cancer patients (for a review, see (O’Farrell et al. 2013)). Executive functioning, processing speed, and memory are domains frequently identified as vulnerable to chemotherapy exposure in this population (Wefel and Schagen 2012). Meta-analyses suggest that CRCI is subtle, may affect a subgroup of patients only, and that, for some, it is a transient phenomenon (Falleti et al. 2005; Stewart et al. 2006). CRCI appears to have the greatest influence on cognitive functioning immediately following treatment to six months post treatment (Jim et al. 2012). However, some studies have found mild impairment years beyond treatment (Ahles et al. 2002; Koppelmans et al. 2012), while others have found pre-chemotherapy impairment in the breast cancer population (Ahles et al. 2007; Wefel et al. 2010), hinting at other contributing factors including the disease itself and highlighting the need for prospective longitudinal study designs.

Neuroimaging studies of chemotherapy-exposed breast cancer patients have started to elucidate the neural underpinnings of CRCI (for reviews, see (McDonald and Saykin 2013; Scherling and Smith 2013)). Research into the neuroanatomical correlates of CRCI has employed voxel-based morphometry (VBM) to explore grey matter compromise in the breast cancer population (Inagaki et al. 2007; Hakamata et al. 2007; Yoshikawa et al. 2005a; Yoshikawa et al. 2005b; McDonald et al. 2010; McDonald et al. 2012a; Conroy et al. 2012; Scherling et al. 2012a; Hosseini et al. 2012; De Ruiter et al. 2011a; Koppelmans et al. 2011). VBM is a technique that enables researchers to make voxel-by-voxel comparisons of images of segmented brain matter volumes between groups of participants in an automated and unbiased manner (Ashburner and Friston 2000; Good et al. 2001). The breast cancer literature suggests that the course of grey matter loss is similar to the course of CRCI. An early, retrospective VBM study found prefrontal and temporal grey matter reductions in a chemotherapy-exposed group four months after exposure; however, these differences were not present when the same study was conducted on a larger group a mean of 4.2 years since chemotherapy exposure (Inagaki et al. 2007). Some studies have found grey matter abnormalities in breast cancer patients at approximately 9.5 years after treatment (De Ruiter et al. 2011a) and 21 years after chemotherapy (Koppelmans et al. 2011), suggesting that a subset of breast cancer patients exposed to chemotherapy are vulnerable to long-term grey matter deficits after chemotherapy exposure. The first prospective VBM study to investigate chemotherapy-related brain matter changes in breast cancer patients found no pre-chemotherapy structural differences between breast cancer patients and healthy controls while conducting a whole-brain analysis (McDonald et al. 2010). One month following treatment, the chemotherapy-exposed group displayed distributed grey matter attenuation that partially recovered one year subsequent to treatment. That was the first study to demonstrate a pattern of grey matter attenuation consistent with the course of cognitive impairment reported in neuropsychological studies, warranting a replication and extension study examining the link between neuropsychological functioning and grey matter disruption in chemotherapy treated breast cancer patients.

To date, only one VBM study has investigated the relationship between grey matter volume and the results of a comprehensive neuropsychological assessment (Conroy et al. 2012). In that retrospective study, grey matter density in the right superior and middle frontal gyri was positively correlated with post-chemotherapy interval. Furthermore, overall neuropsychological performance was positively related to mean grey matter density of these regions. In light of those important findings, and given the cross-sectional design of that study, there exists a need for an increase in longitudinal studies examining grey matter alterations and their relationship with neuropsychological functioning.

In the present study, we employed VBM to measure longitudinal differences in whole-brain grey matter in breast cancer patients exposed to chemotherapy and we examined the relationship of these grey matter alterations to performance on a comprehensive neuropsychological battery. The present work extends a preliminary study conducted by our group that compared pre-chemotherapy volumetric differences between breast cancer patients and healthy controls (Scherling et al. 2012a). Given that current VBM studies suggest that grey matter reductions are most pronounced soon after chemotherapy and partially resolve over time (McDonald et al. 2010; Conroy et al. 2012), it was hypothesized that breast cancer patients would have broadly reduced grey matter volumes following chemotherapy and that some recovery would be observed one year after treatment. We further hypothesized that frontotemporal areas exhibiting grey matter loss would be related to cognitive dysfunction, based on two lines of evidence. First, participants of this study were a subset of participants from a larger neuropsychological study (Collins et al. 2013) that showed a dose-response decline of cognitive functioning. Secondly, previous studies have demonstrated grey matter loss in frontotemporal regions and functional studies (Ferguson et al. 2007; McDonald et al. 2012b; De Ruiter et al. 2011b; Kesler et al. 2011; Kesler et al. 2009; López Zunini et al. 2013) have shown abnormal activations in these areas during executive functioning and memory tasks.

## Materials and method

### Participants

Twenty-three early-stage breast cancer patients and 23 healthy controls matched on age, sex, and education were recruited from the Ottawa Hospital Regional Cancer Centre following patient surgery to remove the cancer, but before patient chemotherapy commencement. Two patients withdrew from the study after treatment. At one year post-treatment, one patient withdrew and another had a recurrence and was excluded from the study. Members of the control group were recruited either by patient nomination or via print and web-based advertisements. The final sample for this study consisted of 19 breast cancer patients and 19 healthy controls. The present sample is a subset of participants from a larger study in which 60 breast cancer patients and their matched controls underwent longitudinal neuropsychological assessment (Collins et al. 2013) with a portion (38%) agreeing to further participate in imaging studies. As part of a larger imaging study, participants performed fMRI tasks related to verbal memory retrieval, response inhibition, and working memory following the structural scan (López Zunini et al. 2013; Scherling et al. 2011; Scherling et al. 2012b).

Clinical and demographic characteristics, including chemotherapy regimens, are listed in Table 1. Inclusion criteria for both groups were: 1) female; 2) no previous history of cancer or chemotherapy; 3) between 18 and 65 years of age at diagnosis; 4) fluent in English; and, 5) minimum of grade 8 education. Potential participants were excluded due to the presence of any of the following: 1) metastasis of disease beyond axillary lymph nodes, 2) neo-adjuvant chemotherapy treatment, 3) serious psychiatric illness, neurological illness, or substance abuse, 4) MRI incompatibilities (e.g. metal implants, claustrophobia). This study was approved by the Ottawa Hospital Research Ethics Board and the University of Ottawa Research Ethics Board.

**Table 1:** **Demographic and clinical characteristics**

	Patients (***n*** = 19)	Controls (***n*** = 19)	***p-value***
Age at baseline (years)	50.2 (8.6)	49.3 (9.0)	0.76
Education			0.66
High School	2	3	
College	8	8	
Undergraduate Degree	5	2	
Graduate Degree	4	6	
Menopausal status at baseline			0.82
Menstruating	8	9	
Perimenopausal	4	2	
Postmenopausal	7	8	
Cancer stage			
I	3	–	
II	13	–	
III	3	–	
Chemotherapy regimen			
FEC-D (six cycles)^1^	12	–	
FEC-D (five cycles)	1		
CD (four cycles)	4	–	
CDOX (four cycles)^2^	2	–	
Type of surgery			
Modified Radical MX	7	–	
Simple MX	1	–	
Segmental MX	3	–	
Lumpectomy	8	–	
Time between (days)			
Surgery to T1 MRI	49.9 (15.2)	–	
T1 MRI to chemo	6.2 (4.9)	–	
End chemo to T2 MRI	32.0 (15.3)	–	
T1 MRI to T2 MRI	128.8 (23.0)	127.0 (25.0)	0.81
T2 MRI to T3 MRI	406.16 (70.3)	449.7 (106.9)	0.15

Mean (SD) or count values are shown. Units are arbitrary unless otherwise specified. FEC-D: fluorouracil + epirubicin + cyclophosphamide + docetaxel; CD: cyclophosphamide + docetaxel; CDOX: cyclophosphamide + doxorubicin; MX: mastectomy. ^1^Two cases with epirubicin and one case with bevacizumab; ^2^one case with paclitaxel.

### Consent

Written informed consent was obtained from all participants for the publication of this study and its accompanying images.

### Neuropsychological assessment

Prior to chemotherapy, following each patient’s chemotherapy cycle, and one year after treatment completion, patients underwent a pencil-and-paper neuropsychological test battery as well as a computerized cognitive test (CNS-Vital Signs (Gualtieri and Johnson 2006; Gualtieri and Johnson 2008)). The traditional neuropsychological tests (Wechsler 1997; Army 1944; Fischer et al. 2001; Rao et al. 1991; Brown 1958; Delis et al. 2001; Brandt and Benedict 2001; Benedict 1997), listed in Table 2, were selected to parallel the cognitive domains covered by the computerized test battery and on the basis of their previously-observed sensitivity to the effects of cancer treatments (Stewart et al. 2008), their established reliability and validity (Wechsler 1997; Delis et al. 2001; Brandt and Benedict 2001; Benedict 1997; Lezak et al. 2004; Strauss et al. 2006), and the recommendations from the International Cognition and Cancer Task Force (Wefel et al. 2011). To mitigate practice effects, raw neuropsychological patient data were converted to standardized scores based on the means and standard deviations of the control group. Four domain-specific cognitive summary scores were computed on rational and empirical grounds: Processing Speed, Working Memory, Verbal Memory, and Visual Memory. Further elaboration of the assessments and the methodology employed to create the cognitive domains used in this study is provided elsewhere (Collins et al. 2013). Neuropsychological data obtained closest to, but not surpassing, MRI data acquisition were used for analysis.

**Table 2:** **Neuropsychological battery organized by cognitive domain**

Processing speed	Working memory	Verbal memory	Visual memory
Digit-Symbol Coding, WAIS-III (Wechsler 1997)	Digit Span, WAIS-III (Wechsler 1997)	Hopkins Verbal Learning Test-Revised (HVLT-R) (Brandt and Benedict 2001)	Brief Visuospatial Memory Test-Revised (BVMT-R) (Benedict 1997)
Symbol Search, WAIS-III (Wechsler 1997)	Letter-Number-Sequencing, WAIS-III (Wechsler 1997)	CNS-VS Verbal Memory Index (Gualtieri and Johnson 2006; Gualtieri and Johnson 2008)	CNS-VS Visual Memory Index (Gualtieri and Johnson 2006; Gualtieri and Johnson 2008)
Trail Making Test A (Trails A) (Army 1944)	Paced Auditory Serial Addition Task (PASAT) (Fischer et al. 2001; Rao et al. 1991)		
Trail Making Test B (Trails B) (Army 1944)	Auditory Consonant Trigrams Test (CCCs) (Brown 1958)		
CNS-VS Processing Speed Index (Gualtieri and Johnson 2006; Gualtieri and Johnson 2008)	Controlled Oral Word Association Test (COWA) (Delis et al. 2001)		
CNS-VS Reaction Time Index (Gualtieri and Johnson 2006; Gualtieri and Johnson 2008)	CNS-VS Flexibility Index (Gualtieri and Johnson 2006; Gualtieri and Johnson 2008)		
	CNS-VS Working Memory Index (Gualtieri and Johnson 2006; Gualtieri and Johnson 2008)		

### Magnetic resonance imaging

MRI data for the patient group were acquired at three time points, with patient data acquired in similar intervals: T1) after surgery but before chemotherapy, radiation, and/or anti-estrogen treatment; T2) approximately one month subsequent to chemotherapy regimen completion; and T3) approximately one year following chemotherapy.

All images were acquired with a 1.5 Tesla Siemens Magnetom Symphony MR scanner. A gradient echo localizer was acquired and used to prescribe a 3D FLASH (Fast Low Angle SHot) spoiled gradient sequence with the following parameters: TR = 629 ms, TE = 15 ms, field of view: 187 × 250 mm, flip angle: 90 degrees, acquisition matrix: 256 × 192, 5 mm thick axial slices, voxel size 1 × 1 × 5 mm.

The 3D data were analyzed using FSL-VBM (Douaud et al. 2007), an ‘optimized’ VBM protocol (Good et al. 2001) implemented in FSL tools (Smith et al. 2004). The brain extraction tool BET (Smith 2002), was used to remove skin and skull. Subsequently, the brain-extracted images were tissue-segmented and the grey matter partial volume images were registered to the MNI152 standard space using non-linear registration (Andersson et al.). The registered images were averaged and flipped along the x-axis to create a symmetric, study-specific grey matter template in order to reduce the effect of inter-subject variability during registration. The native grey matter images were then linearily re-registered to this template and modulated (i.e. divided by the Jabobian of the warp field) to correct for local expansion or contraction due to the non-linear component of the spatial transformation. Smoothing with an isotropic Gaussian kernel with a sigma of 3 mm was applied to the modulated grey matter images.

Next, within-group voxel-wise threshold-free cluster enhancement-based (Smith and Nichols 2008) GLM analyses were conducted using permutation-based non-parametric testing with 5,000 permutations on whole-brain grey matter volumes. Statistical maps of within-group comparisons thresholded at *p* < 0.01 uncorrected for multiple comparisons were used to generate region of interest (ROI) masks. Uncorrected values were used as a means of selecting ROIs for further analysis. A composite whole-brain mask covering all regions of significant differences and 14 masks covering the intersection of these regions and anatomical ROIs were defined with the AAL atlas tool (Tzourio-Mazoyer et al. 2002) using WFU PickAtlas (Maldjian et al. 2004; Maldjian et al. 2003) were created.

### Region of interest analysis

Using R (The R Project for Statistical Computing, http://www.r-project.org), ROI data were compared across time points with Welch’s t-tests and *p* values were adjusted for multiple comparisons using the Benjamini-Hochberg procedure (Benjamini and Hochberg 1995).

### Neuropsychological and demographic data

Differences across neuropsychological scores were investigated with repeated measures ANOVA and Tukey pairwise comparisons. The relationship between grey matter volumes and neuropsychological performance within the patient group was examined with HLM7 (Raudenbush et al. 2011) using a two-level hierarchical linear model (HLM) (Raudenbush and Bryk 2002) with time points nested within patients. Distinct HLM analyses were conducted in order to assess the correlation between each ROI and each cognitive domain. Welch’s two sample t-tests were used to compare all demographic data, except in the case of nominal data where Fisher’s exact tests were used.

## Results

### Sample characteristics

Demographic characteristics are listed in Table 1. The patient group ranged in age from 35 to 64 years and the controls ranged in age from 31 to 61 years. The interval between scanning sessions did not differ between groups (*p* > 0.05, Table 1). For patients, T1 neuropsychological assessments were conducted on average 10.20 days (*SD* = 8.12) before chemotherapy, T2 neuropsychological assessments were conducted on average 17.75 days (*SD* = 7.17) after final chemotherapy exposure, and T3 neuropsychological assessments were completed on average 392.5 days (*SD* = 46.77) following T2 assessments. After T2 and before T3, 10 patients commenced hormonal therapy. Similarly, during this interval 13 patients underwent radiotherapy. Between T2 and T3, all patients that were either menstruating or perimenopausal at T1 became menopausal; however, this status did not change for controls.

### Within-group grey matter changes

**Figure 1:**
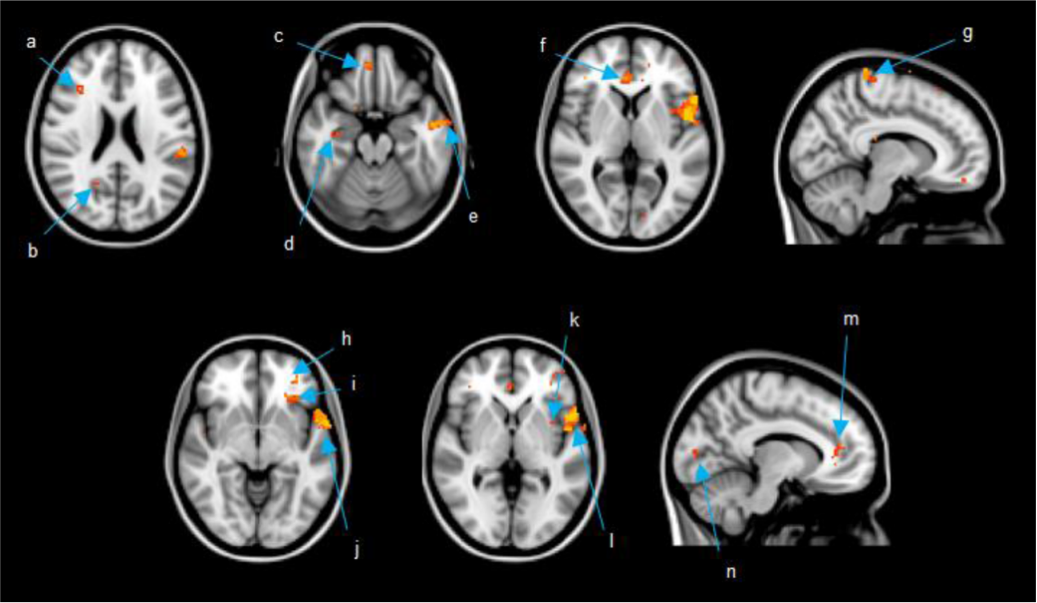
**Images are presented in radiological view where right is the patient’s left side and left is the patient’s right side.** Coordinates presented in MNI space. **a)** middle frontal gyrus (36 28 22); **b)** precuneus (20 -58 25); **c)** gyrus rectus (6 48 -20); **d)** hippocampus (34 -14 -20) **e)** middle temporal gyrus (-58 -4 -20); **f)** anterior cingulate (4 38 5); **g)** paracentral lobule (10 -36 75); **h)** medial orbitofrontal gyrus (-32 44 -5); **i)** inferior orbitofrontal gyrus (-32 28 -5); **j)** superior temporal gyrus (-65 2 -5); **k)** insular cortex (-36 6 0); **l)** inferior frontal operculum (-54 10 0); **m)** anterior cingulate (-10 38 10); **n)** calcarine cortex (-12 -86 5).

Table 3 shows the grey matter volume differences between scans for patients in the composite whole-brain mask and ROIs. At T2 relative to T1, patients showed a reduction of grey matter volume in frontal, temporal, parietal, and occipital regions (Figure 1). There were no areas of increased volume at T2 relative to T1. At one year after chemotherapy relative to T1, significant grey matter reductions were observed in bilateral frontal and temporal regions and all other reductions observed from T1 to T2 were no longer significant. Controls did not show any decrease from T1 to T2; however, they did display an unexpected increase in grey matter volume in the right amygdala from T1 to T2. At T3 relative to T1, this increase was no longer significant; yet, there was a significant increase in grey matter volume in the left lingual gyrus.

**Table 3:** **Longitudinal changes in patient VBM values**

	Pre-chemo to 1-month post (***n*** = 19)			Pre-chemo to 1-year post (***n*** = 19)			1-month post to 1-year post (***n*** = 19)		
Regions	Mean ∆	***T***value	***p***value	Mean ∆	***T***value	***p***value	Mean ∆	***T***value	***p***value
Composite Whole-Brain	-909.08 ± 162.64	-11.70	<0.001	-387.78 ± 164.58	-4.95	<0.001	535.88 ± 186.53	6.04	<0.001
*Left Hemisphere*									
Medial Orbitofrontal Gyrus	-5.85 ± 3.11	-3.94	0.003	-2.52 ± 3.59	-1.474	0.158	3.63 ± 4.17	1.83	0.130
Inferior Orbitofrontal Gyrus	-9.62 ± 4.29	-4.69	<0.001	-3.62 ± 4.75	-1.61	0.125	6.40 ± 4.29	3.13	0.008
Inferior Frontal Operculum	-26.21 ± 11.68	-4.70	<0.001	-15.70 ± 10.29	-3.20	0.007	12.38 ± 14.28	1.82	0.085
Middle Temporal Gyrus	-95.23 ± 40.65	-4.90	<0.001	-48.55 ± 74.59	-1.37	0.188	47.47 ± 65.50	1.52	0.152
Insular Cortex	-20.91 ± 8.48	-5.16	<0.001	-16.14 ± 11.29	-3.00	0.011	4.82 ± 11.29	0.90	0.381
Superior Temporal Gyrus	-79.35 ± 29.94	-5.55	<0.001	-40.30 ± 24.20	-3.50	0.004	42.86 ± 27.16	3.32	0.004
Anterior Cingulate	-15.49 ± 5.14	-6.30	<0.001	-8.87 ± 8.08	-2.31	0.033	5.83 ± 9.04	1.36	0.192
Calcarine Cortex	-3.70 ± 1.80	-4.41	<0.001	-0.43 ± 2.35	-0.39	0.703	3.11 ± -2.18	2.99	0.011
*Right Hemisphere*									
Middle Frontal Gyrus	-8.30 ± 3.70	-4.70	<0.001	-4.97 ± 3.26	-3.20	0.007	3.92 ± 4.52	1.82	0.085
Gyrus Rectus	-29.36 ± 12.97	-4.74	<0.001	-3.99 ± 21.89	-0.38	0.706	24.03 ± 20.51	2.46	0.036
Paracentral Lobule	-34.50 ± 14.91	-4.84	<0.001	-18.34 ± 21.67	-1.78	0.092	17.40 ± -16.53	2.21	0.060
Precuneus	-6.89 ± 2.61	-5.53	<0.001	-5.61 ± 5.84	-2.02	0.089	0.80 ± 6.17	0.27	0.788
Hippocampus	-11.41 ± 3.63	-6.58	<0.001	-7.89 ± 5.71	-2.91	0.014	3.01 ± 5.11	1.24	0.232
Anterior Cingulate	-11.20 ± 4.88	-4.80	<0.001	-4.99 ± 5.74	-1.82	0.100	5.42 ± 6.53	1.74	0.100

Units are mm^3^; Mean ∆: mean difference ± 95% confidence interval.

### Cognitive domain scores

Patient mean scores are presented in Table 4. An analysis of variance for processing speed revealed a significant difference among the time points (*F*(2, 35) = 14.59, *p* < 0.001). A post hoc Tukey test showed that patients scored significantly better at baseline relative to T2 (*p* < 0.001) and T3 (*p* = 0.004). Although processing speed scores improved from T2 to T3, this difference was only marginally significant (*p* = 0.094). Scores on the three other cognitive domains degraded over time; however, the observed differences were not significant.

**Table 4:** **Patient within-group cognitive domain scores ANOVA**

	Time1		Time 2		Time 3		ANOVA		Tukey’s HSD		
	***n*** = 19		***n*** = 19		***n*** = 19		***df*** = 2, 35		T1 to T2	T1 to T3	T2 to T3
Domain	*M*	*SD*	*M*	*SD*	*M*	*SD*	*F*-ratio	*p* value	*p* value	*p* value	*p* value
Processing speed	-0.05	1.03	-0.54	1.29	-0.43	1.19	14.59	<0.001	<0.001	0.004	0.094
Working memory	-0.26	0.71	-0.36	0.70	-0.35	0.72	0.80	0.457	0.421	0.887	0.726
Verbal memory	-0.18	1.14	-0.20	1.25	-0.64	1.44	2.40	0.105	0.993	0.121	0.154
Visual memory	-0.08	0.93	-0.28	0.97	-0.39	1.11	1.39	0.262	0.416	0.259	0.942

Scores represent average z-scores on tests comprising each domain, referenced to the control group mean and standard deviation at the same time point.

### Cognitive functioning and relationship to grey matter volumes

Listed in Table 5 are the correlations between ROI grey matter volumes and the four cognitive domains. Processing speed displayed a positive relationship with the whole-brain composite (*r* = 0.61, p <0.001) and frontal, temporal, and occipital areas. Working memory showed a positive relationship with the left medial orbitofrontal gyrus (*r* = 0.51, p = 0.007) and the right middle frontal gyrus (*r* = 0.42, *p* < 0.05). Visual memory was positively related to grey matter volume in the left inferior frontal operculum (*r* = 0.71, *p* = 0.009) and the right middle frontal gyrus (*r* = 0.71, *p* < 0.009). There was no relationship between grey matter in the ROIs and composite whole-brain with verbal memory.

**Table 5:** **Patient whole brain and ROI grey matter volume correlations with cognitive domains**

Region	Processing speed			Working memory			Verbal memory			Visual memory		
	r	t(18)	***p***value	r	t(18)	***p***value	r	t(18)	***p***value	r	t(18)	***p***value
Composite Whole-Brain	0.61	4.75	<0.001	0.01	0.09	0.933	0.05	0.16	0.877	0.18	0.85	.407
*Left Hemisphere*												
Medial Orbitofrontal Gyrus	0.36	4.27	<0.001	0.51	3.05	0.007	-0.35	-1.20	0.244	0.19	0.88	0.393
Inferior Orbitofrontal Gyrus	0.36	3.91	0.001	0.12	0.75	0.461	-0.63	-1.81	0.088	-0.18	-0.75	0.461
Inferior Frontal Operculum	0.54	3.15	0.006	0.42	2.11	0.049	0.48	1.25	0.229	0.71	2.91	0.009
Middle Temporal Gyrus	0.21	1.29	0.213	-0.02	-0.15	0.883	0.24	0.76	0.457	0.14	0.63	0.540
Insular Cortex	0.44	4.21	<0.001	-0.07	-0.43	0.672	-0.10	-0.27	0.789	0.05	0.21	0.837
Superior Temporal Gyrus	0.56	4.56	<0.001	0.17	0.01	0.948	0.09	0.29	0.775	0.05	0.22	0.829
Anterior Cingulate	0.35	2.25	0.037	0.12	0.91	0.376	0.04	0.11	0.917	0.31	1.39	0.180
Calcarine Cortex	0.26	2.55	0.020	-0.01	-0.09	0.929	0.15	0.48	0.640	0.13	-0.56	0.582
*Right Hemisphere*												
Middle Frontal Gyrus	0.54	3.15	0.006	0.42	2.11	0.049	0.48	1.25	0.229	0.72	2.91	0.009
Gyrus Rectus	0.14	0.94	0.359	0.09	0.56	0.582	0.15	1.25	0.229	0.32	1.50	0.150
Paracentral Lobule	0.58	3.86	0.001	0.14	-0.85	0.408	0.04	0.15	0.886	-0.02	-0.09	0.932
Precuneus	0.19	1.25	0.227	-0.04	-0.38	0.709	0.13	0.39	0.698	0.16	0.59	0.564
Hippocampus	0.26	1.95	0.066	0.10	0.88	0.393	-0.12	-0.40	0.696	-0.08	-0.36	0.721
Anterior Cingulate	0.01	0.09	0.933	0.07	0.56	0.585	-0.15	-0.46	0.653	0.13	0.57	0.577

*n* = 19.

## Discussion

VBM analyses showed diffuse reductions in brain regions of breast cancer patients one month after chemotherapy. This attenuation recovered in nearly half of the regions one year post-chemotherapy. These results provide both evidence of a neural basis for CRCI and optimism for the recovery from the injurious effects that chemotherapy appears to have on the brain.

Our primary hypothesis that grey matter alterations would be more pronounced and distributed shortly after chemotherapy and then partially resolve one year post-chemotherapy was supported. Diffuse grey matter alterations in the patient group approximately one month after chemotherapy exposure is congruent with existing VBM studies that have shown distributed grey matter disruption in the breast cancer population shortly after chemotherapy treatment (Inagaki et al. 2007; McDonald et al. 2010; McDonald et al. 2012a). Additionally, our results support the converging evidence from both structural (Inagaki et al. 2007; McDonald et al. 2010; McDonald et al. 2012a) and functional studies (McDonald et al. 2012b; De Ruiter et al. 2011b; Kesler et al. 2011; Kesler et al. 2009; López Zunini et al. 2013; Silverman et al. 2007) that the frontal lobes appear particularly sensitive to chemotherapy. These findings are important in light of common reports of acute executive function and working memory difficulties in patients subsequent to chemotherapy (Wefel and Schagen 2012) because these cognitive functions are subserved by the frontal lobes (Fletcher and Henson 2001). Our findings of both attenuated grey matter volume in the frontal lobes and the positive relationship between GM reduction in these regions with poorer performance on executive function, working memory, and visual memory strengthen the neuroanatomical evidence of CRCI.

In contrast to one month post-chemotherapy, fewer regions displayed reduced GM at one year post-treatment relative to baseline. Regions displaying persistent grey matter loss remained bilaterally distributed in frontotemporal regions. Enduring frontal grey matter suppression was found in the left anterior cingulate gyrus, left inferior frontal operculum, and right middle frontal gyrus, congruent with a previous report of chronic frontal insult in chemotherapy-treated breast cancer patients (McDonald et al. 2010). The region of the left superior temporal gyrus and left insula that had pronounced reduction at T2 relative to T1 (see Figure 1) did not fully resolve. The superior temporal gyrus and insula share efferent and afferent connections (Flynn 1999) and this may help explain the concomitant insults to these regions observed in the present study. Interestingly, the right hippocampus displayed reduced grey matter, concordant with previous studies that have demonstrated prolonged hippocampal compromise (McDonald et al. 2010; Kesler et al. 2013; Bergouignan et al. 2011), but in contrast to a study by Yoshikawa et al. (Yoshikawa et al. 2005a) that failed to find hippocampal insult.

The diffuse nature of grey matter modulation observed in this and similar studies, along with some of the regional volume loss discordance across extant grey matter studies of chemotherapy in breast cancer, may be attributable to the inclusion of heterogeneous chemotherapy regimens. Although nearly all cytostatic agents have been associated with neurobiological effects, the mechanisms and outcomes vary across treatments (for a review, see (Seigers et al. 2013)). Some chemotherapeutic agents, such as methotrexate, 5-fluorouracil, and cyclophosphamide, appear to have direct cytotoxic effects via their ability to penetrate the blood-brain-barrier (BBB) (Dietrich 2010); however, other agents appear to have indirect effects due to their inability to cross the BBB. A recent study by Kesler et al. (Kesler et al. 2013) suggests that elevated pro-inflammatory cytokine expression, as seen in BBB impermeable agents like doxorubicin, may have direct and indirect injurious effects on brain structures. Our present study included both BBB permeable and impermeable chemotherapeutic agents (see Table 1). It will be important for future studies to tease apart the differential effects of various chemotherapy treatments on the brain.

Our secondary hypothesis that grey matter attenuation would be related to cognitive functioning was supported. Although processing speed was positively related to distributed grey matter volumes, the association was observed predominantly in frontotemporal regions. This included the left insula and a portion of prefrontal areas with which the insula shares bidirectional connections (Flynn 1999), specifically, the left medial and inferior regions of the orbitofrontal cortex (OFC) and the left inferior frontal operculum. The insula is critical for neural communication between the prefrontal cortex and more posterior regions (Augustine 1996) and disruption to the insula and associated regions may underlie some of the cognitive difficulties expressed by chemotherapy-exposed breast cancer patients.

Interestingly, processing speed and working memory were positively correlated with grey matter volume in the medial orbitofrontal gyrus. This is a notable finding given the clinical implications. Previous studies have associated the OFC with a range of cognitive processes including decision making (Plassmann et al. 2010), emotion (Rolls & Grabenhorst 2008), and response inhibition (Horn et al. 2003). Grey matter loss in this region of the OFC was significant for patients between pre-chemotherapy exposure and one month post-treatment, consistent with the pattern of decreased cognitive performance during the same interval. Our findings underscore the extensive impact that exposure to chemotherapy may have on breast cancer patients. In light of our results, work showing that the OFC is involved in processes beyond the cognitive domains selected in our study points to a potential for chemotherapy exposure to adversely impact the lives of breast cancer patients more widely than suggested by our results alone.

The strengths of this study include its longitudinal design and the administration of a comprehensive battery of objective neuropsychological measures that covered a broad range of cognitive processes. We acknowledge that there are limitations to our study that necessitate a degree of caution when interpreting our findings. Primarily, we did not include a chemotherapy-naïve comparison group and, consequently, we could not control for the potential influence of cancer-related factors. Existing VBM studies that have included both a healthy control group and a chemotherapy-naïve breast cancer control group have found no within-group grey matter differences in these control groups in contrast to the decline observed in chemotherapy-exposed breast cancer patients (McDonald et al. 2010; McDonald et al. 2012a), suggesting that grey matter alterations may stem from chemotherapy exposure.

The number of treatment cycles and types of surgery varied across breast cancer patients (see Table 1). Given our limited sample size, we could not control for the influence of these potentially confounding factors. Currently, one cross-sectional study has examined the effects of a heterogeneous chemotherapy regimen on grey matter volumes in a large sample (Koppelmans et al. 2011); it will be important for future longitudinal investigations to employ a similar approach. Treatment-induced menopausal symptoms co-occur with cognitive impairment following chemotherapy in breast cancer patients (Fan et al. 2005). Although our patients and controls were closely matched at baseline, at T3 relative to T2 all patients were menopausal while controls remained unchanged. As a result, we were unable to control for the effects of menopausal status. From time T2 to T3, some patients received radiotherapy or commenced hormonal therapy. Reports in the literature suggest that these treatments may perturb cognition (Quesnel et al. 2009; Bender et al. 2001). However, despite their administration, an overall improvement in GM volumes was observed from T2 to T3, suggesting that these therapies may have had a negligible effect.

In summary, the present study demonstrated grey matter volume loss in diffuse brain regions in breast cancer patients one month following chemotherapy treatment. One year following treatment, grey matter was partially recovered. Grey matter volumes were related to cognitive performance in the domains of processing speed, working memory, and visual memory. Cognitive dysfunction was found to follow a similar course to grey matter changes, particularly in the domain of processing speed. This study strengthens the evidence for the relationship between brain alterations and objectively measured cognitive difficulties in breast cancer patients exposed to chemotherapy. In addition to the burden of being diagnosed with a life-threatening disease, breast cancer patients must contend with potential adverse side effects of treatment. Cognitive and neurophysiological alterations touch many areas of survivors’ lives, warranting future research to further elucidate the mechanisms of CRCI and to improve breast cancer patients’ quality of life.

### Ethical standards

The experiments of this study comply with the current laws of Canada.
